# A curated benchmark for cofolding models on kinase conformational states

**DOI:** 10.1038/s44386-026-00068-z

**Published:** 2026-09-02

**Authors:** Kunyang Sun, Teresa Head-Gordon

**Affiliations:** 1 Kenneth S. Pitzer Theory Center and Department of Chemistry, University of California, Berkeley, 94720 CA USA; 2 Department of Bioengineering, University of California, Berkeley, 94720 CA USA; 3 Department of Chemical and Biomolecular Engineering, University of California, Berkeley, 94720 CA USA

**Keywords:** Biochemistry, Biophysics, Computational biology and bioinformatics, Drug discovery, Structural biology

## Abstract

Protein kinases are critical drug targets, requiring therapeutics that can modulate their active and inactive conformational states. While cofolding models can generate global folds directly from kinase sequences and ligand SMILES strings, these models have not yet been tested on their ability to recover ligand-induced-fit conformational states of the kinase proteins. Here, we introduce KinConfBench, a curated benchmark of 2225 high-quality human kinase chains to evaluate the ability of four state-of-the-art cofolding models—Boltz-2, Chai-1, Protenix, and RoseTTAFold-All-Atom—to recover both canonical and rare conformational states. We show that geometric success metrics of a ligand pose in the active site do not correlate strongly with the correct kinase conformational state, motivating a new set of dynamical benchmarks for assessing cofolding models. While all four cofolding models achieve ~60–80% prediction accuracy for kinase conformational classification, they exhibit severe mode collapse when performing multiple inferences, show negligible structural diversity in sampling induced-fit motions, and display a prevalent “apo-drift” in which most cofolding models predominantly predict the kinase to be in its ligand-free state. Our results highlight that capturing ligand-induced protein conformational diversity, not just geometric fit, is critical for next-generation structure-based drug discovery.

## Introduction

Highly accurate data-driven protein structure prediction models^1–7^ have become powerful tools for structural biology. By combining evolutionary information with structure-based training using the Protein Data Bank (PDB)^8^, these models have allowed researchers to generate plausible protein structural hypotheses from sequence, especially for proteins that are not structurally enabled by experiment. Inspired by this advancement, new directions have opened up to extend the framework beyond single-chain proteins, predicting protein complexes with other proteins^9,10^ or biologics^11–18^. Consequently, a new class of “cofolding” models has emerged, designed to predict structures for biomolecular complexes including proteins, DNAs, RNAs, post-translational modifications (PTMs), ions, small molecules, and other modalities critical for drug discovery. Models such as RoseTTAFold-All-Atom^13^, AlphaFold3^12^, Chai-1^14^, Protenix^17^, Boltz 1 and 2^15,16^, and NeuralPLexer3^18^, have shown promise in accelerating structure-based drug discovery (SBDD) campaigns by generating structural hypotheses at a reasonable computational cost.

Despite their rapid adoption, data-driven cofolding models suffer from distinct generalization issues. For instance, Škrinjar et al.^19^ observe that these models exhibit significantly lower success rates and confidence scores when tasked with systems outside their training distribution. Beyond these failures in low-data regimes, even predictions with high confidence scores can exhibit physically invalid steric clashes or distorted ligand geometries that fail the benchmark evaluations by the PoseBusters suite^20^. Moreover, recent benchmarking efforts have attempted to probe limitations in other regimes such as covalent modifications^21^, PROTACs^22^, and molecular glues^23^. Besides these emerging and less common modalities, even within the well-represented kinase complexes, Nittinger et al.^24^ reveal that cofolding models still struggle to place allosteric ligands into their allosteric pockets due to the overrepresentation of orthosteric ligands in the PDB training data. In summary, these studies expose the weaknesses of the current cofolding models, collectively suggesting directions for improvements to the next-generation cofolding models.

However, while these benchmarks successfully identify domain-specific challenges, they largely overlook a more fundamental requirement of modern SBDD: the capacity for ligand-modulated conformational reasoning. As detailed above, current evaluations remain predominantly ligand-centric, prioritizing the geometric placement of the small molecule through mostly pocket-aligned ligand Root Mean Square Deviation (RMSD) and overall global structural confidence in the protein fold. While these metrics are excellent starting points to verify basic structural integrity, they treat the protein receptor as a static scaffold rather than a dynamic landscape^25^. In practical drug discovery, identifying the correct global fold is necessary but not sufficient; successful lead optimization requires capturing the specific induced-fit conformational states dictated by ligand binding. Without a focused assessment of this conformational response, there remains a critical gap in understanding whether cofolding models can truly drive rational drug design.

The human kinase family presents an ideal paradigm to address this gap, given its strong representation in the PDB, its importance in therapeutic discovery^26–28^, and the inherently dynamic nature of the kinase conformational landscape^29–31^. Functioning as molecular switches, kinases toggle between active and inactive states via subtle rearrangements of the activation loop (DFG motif) and the *α*C-helix^32,33^. Because these states differ sharply in how readily they crystallize, the resulting structural record is highly imbalanced, as the canonical active DFGin-BLAminus conformation dominates the PDB, while inactive or transient states are comparatively scarce^33^. A model trained to reproduce these statistics would, by default, be expected to favor the common canonical conformation and underrepresent rarer states, making kinases not only a data-rich but genuinely demanding test of whether a model can biophysically reason about ligand-induced conformational change or merely reproduce the dominant training prior. This task is important because the utility of a kinase structure in drug discovery is defined by these specific conformational states, which dictate the kinetics and selectivity of inhibitor binding, distinguishing Type I from Type II inhibitors^34^. Consequently, it is imperative and timely to evaluate the capacity of cofolding models to provide biophysically valid structural hypotheses, specifically by quantifying how well they capture the kinase conformational landscape and generalize across unseen ligand-induced states for drug discovery.

In this work, we introduce KinConfBench, a curated benchmark of 2225 high-quality human kinase chains representing a total of 1420 unique kinase systems (43 unique apo targets and 1377 unique holo complexes), designed to shift the evaluation focus from ligand RMSD to protein-ligand conformational fidelity. To rigorously quantify these states, we employ the structural nomenclature developed by Modi and Dunbrack^33^, labeling kinase chains with categorical structural features to capture specific active and inactive conformations. Using this framework, we evaluate four widely used open-source cofolding models—Boltz-2^16^, Chai-1^14^, Protenix^17^, and RoseTTAFold-All-Atom (RFAA)^13^—on their capability to recover experimentally observed kinase states in the presence of bound small-molecule ligands. The resulting metrics show that high confidence in the active site geometry does not ensure high confidence in predicting the kinase conformational states, especially when ligands perturb the kinase away from common templates of the global fold. We further document a critical generalization gap on kinase holo-apo pairs. Across all three diffusion-based models (Boltz-2, Chai-1, and Protenix), predictions default to the apo state for a large fraction of holo complexes even when those complexes are in the training set. Furthermore, this phenomenon of apo drift increases for complexes deposited after the training cutoff, indicating that the models are anchored to the dominant conformational prior of the training distribution rather than capturing the induced-fit response of the specific ligand. KinConfBench therefore foregrounds protein conformational correctness, not ligand RMSD alone, as the quantities that ultimately need to be ranked and interpreted to make progress in SBDD campaigns.

## Results

### The KinConfBench conformational benchmarks

**Fig. 1:**
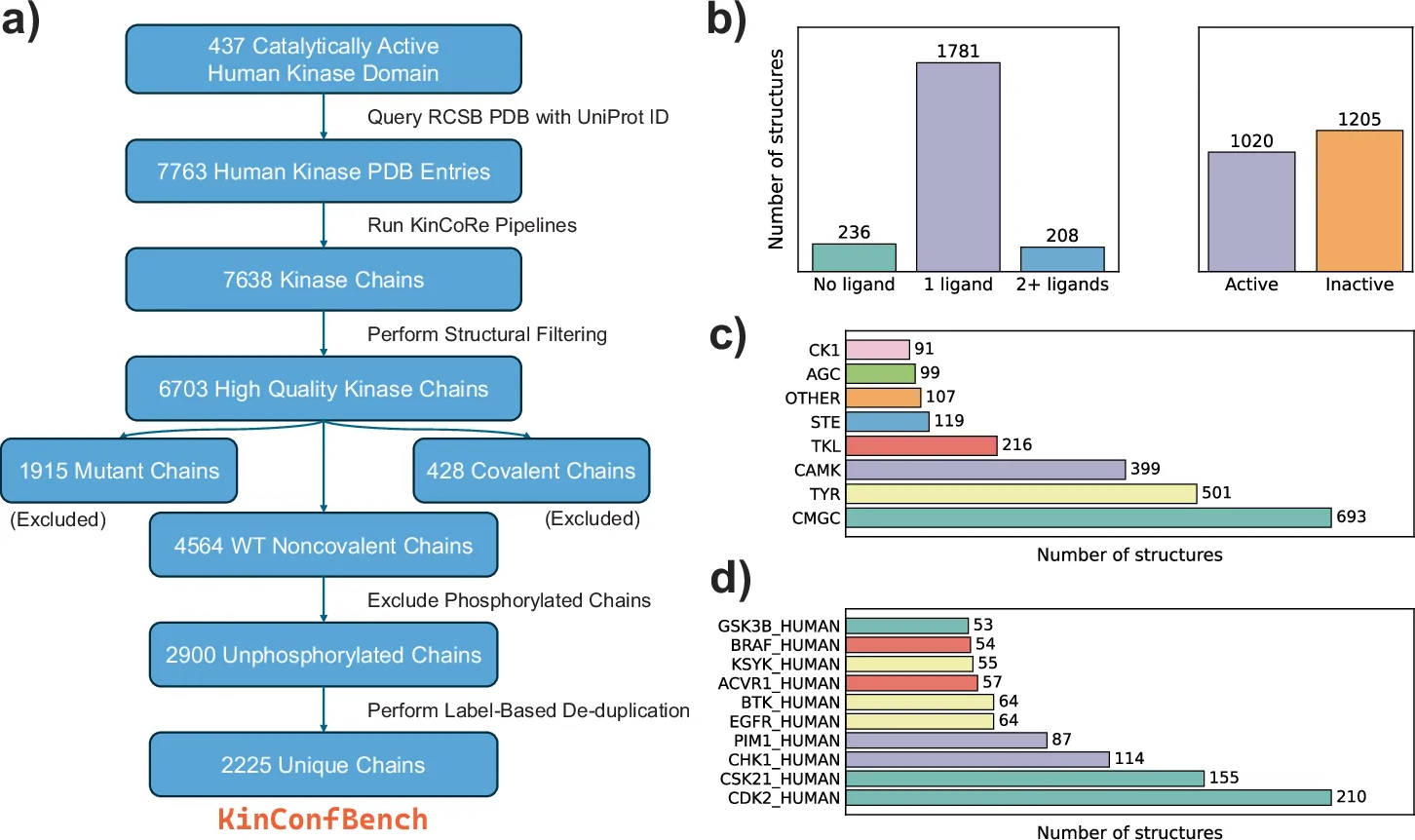
Overview of the KinConfBench curation pipeline and its data distribution. **a** Schematic of the curation workflow, detailing the sequence of filtering and labeling steps. **b** Distribution of ligand counts per kinase chain and their corresponding conformational activity. **c** Taxonomic distribution of kinase chains categorized by major kinase groups. Details for acronyms can be found in Methods. **d** Representation of the top 10 most frequent kinase genes in the dataset, colored by their kinase family.

To rigorously evaluate the capability of cofolding models to capture kinase conformational states, we curate KinConfBench, a high-quality benchmarking dataset derived from the RCSB PDB^8^ as shown in Fig. 1. To construct a comprehensive benchmarking dataset, we first identify 437 catalytically active human kinase domains and their corresponding UniProt^35^ identifiers, following the classification established by Gizzio et al^36^. These UniProt IDs are then used to query the RCSB PDB^8^ (snapshot 2025-08-01) to retrieve all associated structural entries. By prioritizing this UniProt-centric approach over broader sequence-similarity searches, we ensure the specific retrieval of human kinase domains while minimizing potential contamination from non-human homologs. This initial search yields a raw dataset of 7763 unique PDB entries, each containing at least one relevant kinase chain.

To assign conformational labels to the selected kinase PDB entries, we process all chains from the identified entries using the KinCoRe software suite^36,37^. KinCoRe categorizes kinase conformations by extracting and classifying key structural features into different conformational groups. There are a total of eight categorical labels: a spatial label that captures the orientation of the DFG motif; a dihedral label that captures the torsion angle distribution of the X-DFG motif; a helix label that captures the orientation of the *α*C-helix; a salt bridge label that captures the orientation of a conserved kinase salt bridge; N-terminus and C-terminus activation loop labels that capture the orientations of activation loops for both termini; a spine label that captures the orientation of the kinase regulatory spine; and finally, the activity label that assigns whether the kinase structure is in an active or inactive form based on the previous labels. More details on the specific labeling format are provided in the Methods Section.

Following the labeling process, we apply a stringent filtering protocol to exclude structures with missing atomic coordinates in critical functional regions that have “None” annotations in the KinCoRe output. Specifically, kinase chains are discarded if they have “None” values for either the spatial label or the helix label due to missing residues or falling out of predefined structural categories^37^. This rigorous screening for structural motif completeness ensures that our benchmark is based on high-fidelity structural data, resulting in a refined set of 7638 kinase chains.

To ensure stringent data quality control, we excluded chains with a resolution worse than 4.5 Å or those determined by NMR spectroscopy, following the protocol established by NeuralPLexer^11^. Furthermore, for genes encoding both catalytically active and pseudokinase domains, such as members of the Janus Kinase family, we use the reference sequences of the active kinase domains extracted from UniProt to selectively retain only the catalytic units while excluding pseudokinase domains. This final filtering stage yields a high-quality set of 6703 kinase chains.

The filtered dataset is partitioned into three primary categories: mutants (1915 chains), covalent complexes (428 chains), and wild-type (WT) noncovalent complexes (4564 chains). For this study, we focus exclusively on the unphosphorylated subset of the WT noncovalent entries comprising 2900 chains to benchmark co-folding model performances on canonical kinase sequences. To refine this subset, we remove trivial redundancies, defined as entries sharing identical sequences (and ligand SMILES, where applicable) alongside identical KinCoRe conformational labels. Nevertheless, we preserve meaningful structural diversity by retaining any entry that exhibits at least one unique KinCoRe label relative to others of the same condition. This logic is applied both to apo chains of the same gene and to holo complexes featuring the same protein-ligand combination, ensuring that our benchmark accounts for the multi-state nature of kinase domains. By prioritizing these distinct conformational states, we collect the final 2225 structurally unique kinase chains for cofolding model benchmarking. This entire curation process is illustrated in Fig. 1a.

To investigate the general distribution of the selected unique kinase chains, we perform further analysis based on their gene properties and KinCoRe labels. As shown in Fig. 1b, the dataset is primarily composed of single-ligand holo complexes (~80%), with apo structures and multi-ligand complexes each accounting for ~10%. This composition closely mirrors the broader distribution of kinases in the PDB, reflecting the data landscape encountered by co-folding models during training. Furthermore, while there appears to be a balanced global distribution between active and inactive states across the entire dataset, it is worth noting that this ratio can vary quite significantly at an individual gene level.

In terms of the kinase family distribution, we take the Manning classification system^38^ and find that CMGC, TYR, CAMK, and TKL groups dominate the dataset, as these families represent areas of intense therapeutic interest^26–28,39^. Indeed, the ten most frequently occurring genes in our dataset belong to these four groups (Fig. 1c, d). Further distributional metrics, including ligand and KinCoRe label frequencies, are detailed in Fig. S1. We observe a high prevalence of DFG-in and BLAminus labels, alongside a majority of Type I ligands that align with KinCoRe server results as shown in Table S1. This observation indicates a persistent bias in the PDB toward holo complexes where the ligands occupy the ATP-binding pocket in catalytically active states, as the DFG-in/BLAminus configuration is a structural prerequisite for kinase activity^33^.

After filtering for experimental resolution, sequence completeness, and distinct functional domain labels, the final curated dataset of 2225 kinase chains is comprised of 236 unique apo chains representing 43 unique gene targets, and 1989 unique holo chains with 1377 involving unique protein-ligand pairs, ensuring broad coverage of the human kinome.

### Evaluation of cofolding models against KinConfBench

Protein kinases are highly dynamic molecular machines that transition between multiple active and inactive isoforms to perform their function^29–31^. Molecular effectors such as small drug molecules introduce new interactions that can shift the thermodynamic equilibrium among these states. For instance, Type II inhibitors specifically recognize and stabilize the inactive “DFG-out” conformation by binding to an adjacent, allosteric hydrophobic pocket^40,41^. By establishing favorable interactions within this newly exposed site, the ligand significantly lowers the potential energy of the inactive state relative to the active one^42^, and the kinase becomes thermodynamically trapped in this non-functional shape, effectively halting its catalytic activity. Therefore, in the following sections, we evaluate both the geometric accuracy of ligand placement and the models’ ability to capture the correct kinase conformational state. This benchmark allows us to determine whether current cofolding models can truly simulate the structural response of a kinase relative to ligand binding, rather than just predicting a static pocket fit.

#### From geometric filters to functional conformations

Generating valid and conformationally diverse protein folded states is a fundamental prerequisite for the meaningful use of cofolding models. To characterize structural features, we utilize KinCoRe, which assigns each kinase protein structure to spatial and dihedral label combinations (Table S1)^33^. By applying the KinCoRe labels to every prediction, we establish a “ground truth” based on the categorical orientation of the DFG motif and the *α*C-helix, yielding a structural metric that is more functionally relevant than standard backbone RMSD. When mapping our inference results against these established clusters, we found that the vast majority of cofolded structures match the 8 conformational categories of the KinConfBench chains defined by their spatial and dihedral labels (Table S2), aside from a small fraction of structures that receive “None” labels and fall into the other four possible classifications. This observation confirms that most tested cofolding models possess the baseline biophysical reasoning necessary to generate structurally plausible kinase conformations.

Beyond the biophysical validity of the protein fold, a cofolding model must place the ligand correctly into the pocket, which we characterize using three structural metrics. The global fold quality, captured by the local Distance Difference Test on C*α* (lDDT-C*α*), summarizes the C*α* agreement of the predicted complex to the experimental structure, where higher values indicate a closer global backbone match. The pocket-focused geometry metric, the local Distance Difference Test on protein-ligand interaction (lDDT-PLI), emphasizes the agreement of the binding site and ligand-adjacent protein atoms compared to experimental values. Finally, the protein-aligned ligand heavy-atom RMSD (Ligand RMSD) indicates the agreement of the modeled ligand pose with the crystal pose. The overall distribution of these geometric values for each cofolding method is captured in Fig. S2.

We define a geometrically successful prediction as one with global fold lDDT-C*α* ≥ 0.7, binding pocket lDDT-PLI ≥ 0.8, and ligand RMSD < 2.0 Å following the definition of previous work^2,19^. From the initial pool of 1420 protein-ligand kinase systems, geometric filtering identifies 1125 valid predictions for Boltz-2, 1099 for Chai-1, 1,075 for Protenix, and 451 for RFAA, where each cofolding model produces at least one geometrically valid pose out of 20 samples for downstream analysis. In Table S3, we show that, first of all, the removal of geometrically invalid samples eliminates all data that previously had a ‘None’ value. Besides that, the KinCoRe spatial and dihedral labels for geometrically valid samples have a higher percentage of the canonical DFGin-BLAminus and DFGout-BBAminus conformations, while showing a reduction in all other categories for the three diffusion-based cofolding methods. In contrast, RFAA exhibits no substantial changes across categories, demonstrating that geometric filtering selectively reduces the predictions of Boltz-2, Chai-1, and Protenix that fall into rare, non-canonical conformations.

**Fig. 2:**
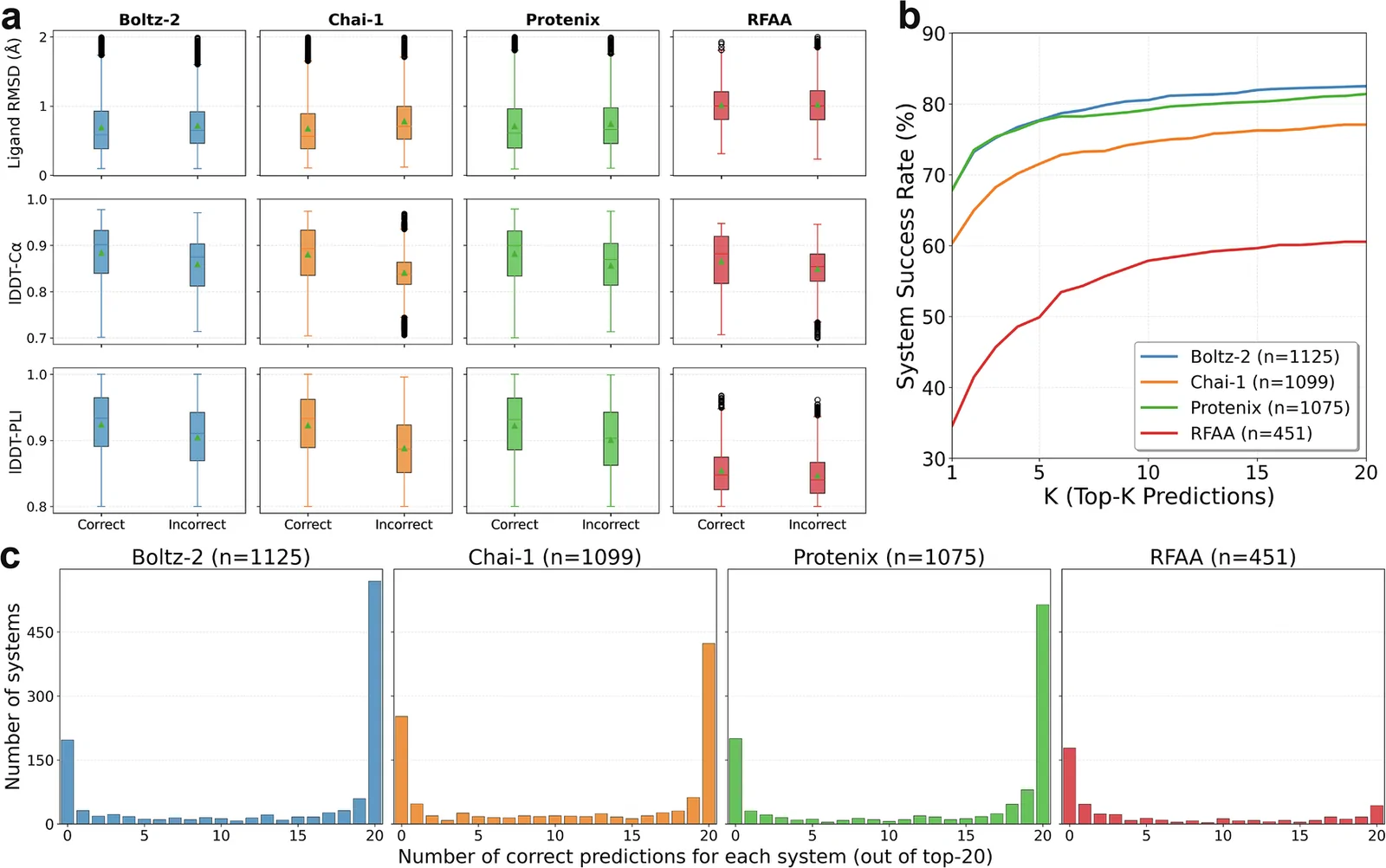
Correct and incorrect KinCoRe classification for all four cofolding model predictions passing geometric filters. **a** Cofolding model performance on geometric predictions (lDDT-C*α* ≥ 0.7, lDDT-PLI ≥ 0.8, ligand RMSD < 2.0 Å) comparing samples with all eight KinCoRe labels being correct versus incorrect. To account for intrinsic structural flexibility where a single experimental entry may capture multiple conformational states, we consider a prediction successful if it matches any valid set of ground truth labels associated with that protein-ligand complex. **b** Success rate of each cofolding model versus a correct ranked prediction among the top-*K* by confidence. For each target, we evaluate an ensemble of *N* = 20 generated structures, ranked by their pLDDT confidence scores. **c** Histogram of the count of top-20 predictions that match all KinCoRe labels of their corresponding experimental structures. The *x* axis is the number of correct predictions in the ranked ensemble (0-20), and the *y*-axis is how many of the geometrically correct kinase systems fall in each bin for each cofolding method.

However, a geometrically successful binding pose does not ensure that the cofolding model has captured the correct induced conformational state. Specifically, a prediction is only considered “correct” if all eight KinCoRe labels match those of the experimental structures. Figure 2a shows the relation of the three geometric metrics (lDDT-C*α*, lDDT-PLI, and Ligand RMSD) to KinCoRe label correctness across all models. While correct conformational assignments skew better across the geometric metrics, the overlap with incorrect functional assignment remains large. The side-by-side comparison among the cofolding models highlights that mislabeled states can still achieve strong ligand-centric structural scores around the binding pocket, indicating that geometry alone is an incomplete proxy for robust cofolding prediction.

Figure 2b shows each co-folding model’s success versus *K*, defined as the presence of at least one correct functional state among the top-*K* predictions ranked by model confidence. Both Boltz-2 and Protenix lead at Top-1 (67.9% and 67.8%, respectively) compared to Chai-1 (60.3%), followed by RFAA (34.6%). While moving from Top-1 to Top-20 adds roughly 14-17% for the three diffusion models, RFAA improves the most, gaining ~26%, rising from 34.6% to 60.5%. Despite this gain, RFAA’s absolute accuracy falls short, indicating the performance advantages of diffusion-based cofolding models in correctly predicting kinase conformations.

In addition, this upward trend suggests that generating additional samples can occasionally recover the correct conformational state even when the highest-confidence pose is incorrect. Nevertheless, despite this overall increase in accuracy, Fig. 2c reveals that the predictions of the three high-performing cofolding models are largely bimodal, since roughly two-thirds of the predicted kinase-ligand systems result in either entirely successful or entirely failed predictions across the 20 inferences. RFAA, by contrast, is less distinctly bimodal due to its lower baseline performance, yet it follows a similar pattern where predictions for a given system are typically all correct or all incorrect. This binary outcome profile strongly suggests ensemble mode collapse rather than a graded representation of uncertainty across the twenty generated samples for all cofolding models.

**Fig. 3:**
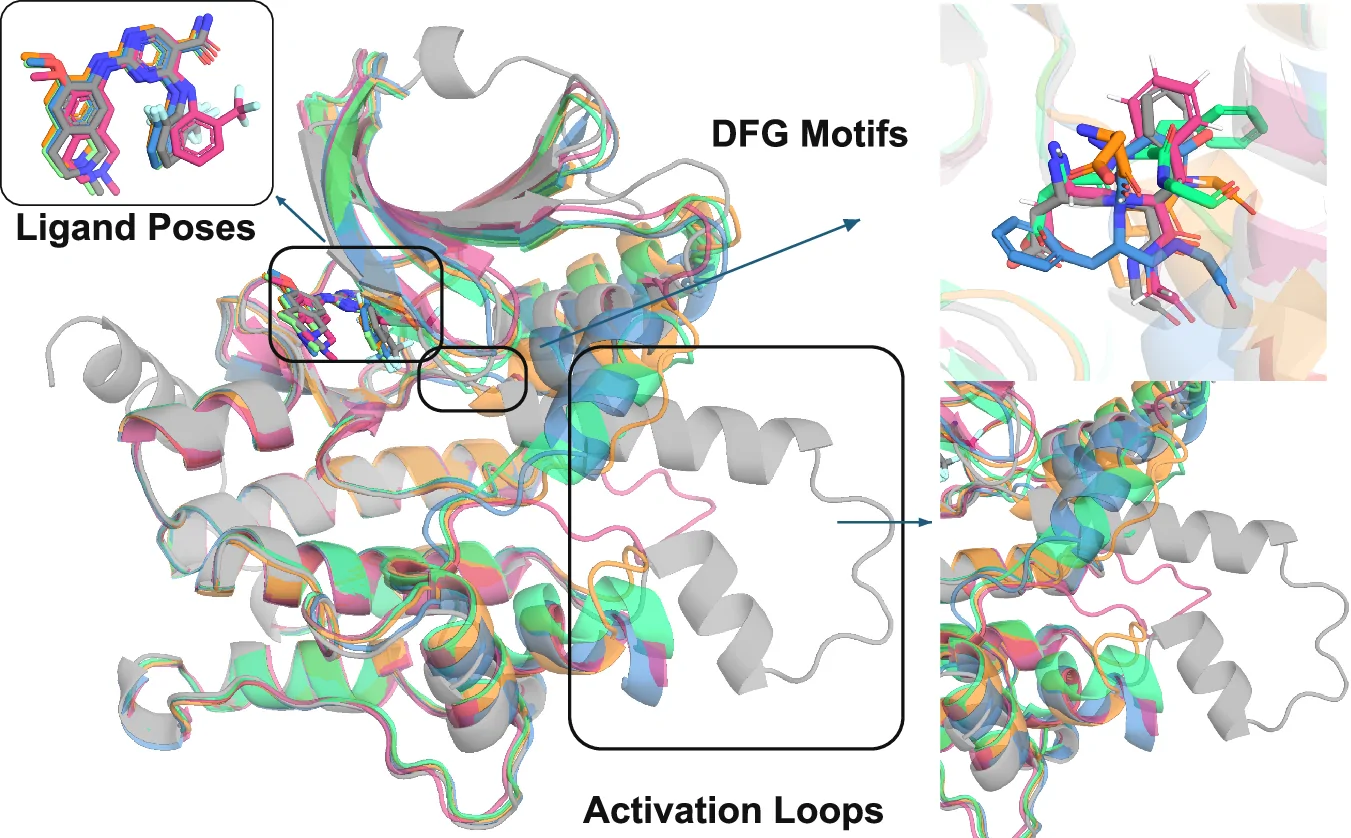
Induced-fit failure modes for MAP4K1 (7M0M). Superposition of the experimental Type I diaminopyrimidine carboxamide complex (gray) with representative Boltz-2 (blue), Chai-1 (orange), Protenix (green), and RFAA (red) predictions evaluated in KinConfBench. The experimental structure adopts DFG-in, BLAminus packing with an inward *α*C-helix and an extended, outward-biased activation loop. (Top right) DFG region; (bottom right) activation loop. Although ligand placements are perfect across models, the four evaluated cofolding predictions miss the cooperative loop sampled in the PDB, collapsing instead toward structures that are conformationally mislabeled relative to 7M0M.

Table S4 contains the 152 kinase-ligand systems where all cofolding models fail to generate the correct conformation, providing an important benchmark for future cofolding model improvements. Figure 3 highlights MAP4K1/YK1 (PDB ID: 7M0M)^43^ as a representative hard case where all cofolding model predictions fail to obtain an induced-fit to the correct conformational state. This observation shows that even when pocket metrics and ligand poses look favorable, Boltz-2, Chai-1, Protenix, and RFAA often jointly settle into an alternate spatial and dihedral configuration that disagrees with the experimentally observed loop excursion, underscoring why ligand RMSD alone can mask protein-state errors.

#### Ensemble diversity and resolution of complex targets

KinCoRe labels establish whether a model satisfies the mechanistic requirements of a kinase state, such as satisfying the DFG and *α*C-helix motifs, but categorical success does not capture the nuances of intra-basin exploration. Hence, we extend our analysis beyond discrete accuracy to measure the structural dispersion within ensembles that are already deemed functionally correct.

To quantify conformational diversity, we use the same geometric descriptors that define the KinCoRe labels, calculated on a continuous scale for atomic distances and backbone or side-chain dihedral angles, as described in Methods. For this benchmark, we focus on systems where each cofolding model produces at least ten all-correct structures according to KinCoRe within the top-20 predictions, ensuring that every evaluated method contributes a comparably populated success set based on classification. Overall, this diversity criterion leaves us with 780 systems for Boltz-2, 666 for Chai-1, 754 for Protenix, and 138 for RFAA. Figure S3 summarizes the distribution of the number of correct predictions on this extracted diversity subset for each cofolding method, where we still observe that most of the correct predictions center around having all 20 correct predictions.

**Fig. 4:**
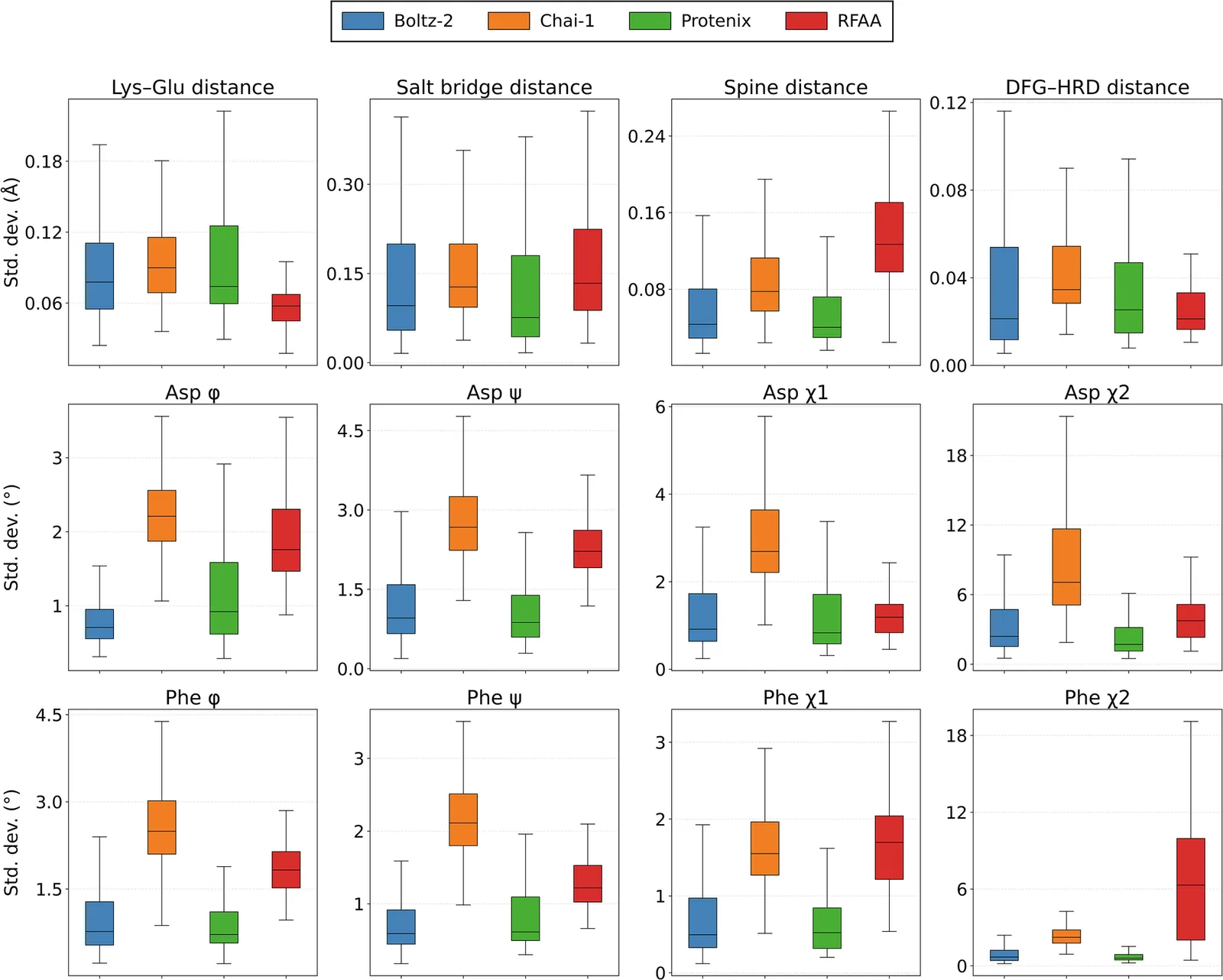
Distance and torsion angle ensemble diversity among correct KinCoRe predictions across cofolding models. For each system in the diversity subset from KinConfBench, we retain only cofolding predictions that are all-label correct, then measure the distance and dihedral angle standard deviations across those correct poses. Overall, all cofolding models have little structural diversity. Panels compare Boltz-2 (blue, *n* = 780), Chai-1 (orange, *n* = 666), Protenix (green, *n* = 754), and RFAA (red, *n* = 138).

Figure 4 shows the standard deviation in distance and torsion angle metrics around their average values across the 20-member ensembles for all four cofolding models. While Fig. S4 demonstrates that all cofolding models except RFAA capture the correct average distance and angle values compared to experiment, all models produce very small structural deviations of ~ 0.1 Å for the distance metrics and <5° of average angle exploration except for Chai-1’s predictions on Asp *χ*_2_ and RFAA’s predictions on Phe *χ*_2_. To contextualize these results, root mean square fluctuations of ~1–2 Å and backbone or side-chain dihedral angle ranges of 5° to 30° define the statistical fluctuations of stable protein conformations^44,45^, while significantly higher values would be needed to indicate transitions between distinct conformational states. Hence, while the Chai-1 and RFAA models show greater exploration within the correct structural basin compared to Boltz-2 and Protenix, on an absolute scale the conformational diversity is still quite limited, consistent with previous ligand-centric analysis of cofolding models^19^.

#### Benchmarking apo-to-holo conformational shift

In structure-based drug design, ligand binding represents a thermodynamic perturbation of the protein’s native conformational landscape. We conceptualize the apo (ligand-free) distribution as a cofolding model’s learned default energy landscape, whereas the introduced ligand acts as a physical modulator that distorts this baseline to stabilize a distinct holo conformation. If a cofolding model learns the underlying physics of molecular recognition, it successfully predicts this specific ligand-induced apo-to-holo conformational shift. Conversely, if a cofolding model relies heavily on memorization, it would be expected to fail to generalize to an apo-to-holo conformational transition for novel ligand effectors unseen by the model during training, effectively “drifting” back to the unperturbed, default apo state.

To test this hypothesis, we perform a comparative analysis against the ground-truth holo and apo state pairs. We extract kinase systems from KinConfBench that possess resolved structures for both states, restricting our analysis to systems where the ligand induces a distinct structural change defined as a different set of KinCoRe functional labels compared to ligand-free structures. For each cofolding model, we define training and test sets based on a time split relevant to each model: a holo ligand combination counts as part of the test set if it first appears in the PDB only after that method’s training data deposition cutoff: Boltz-2 (2023-06-01); Chai-1 (2021-01-12); Protenix (2021-09-30); and RFAA (2020-04-30). All apo-state structures are found in the training data for all cofolding models.

For each of the kinase system pairs, we generate an ensemble of 20 structures using the holo kinase-ligand pair as input for each cofolding model. After filtering for geometrically valid samples following the geometric criteria defined above, we classify their predictions according to:

- Holo only: At least one structure in the generated ensemble exactly matches the KinCoRe labels of the ground-truth holo reference and 0 matches the KinCoRe labels of the ground-truth apo reference. This metric quantifies the model’s ability to correctly predict the ligand-induced state.
- Apo only (drift): At least one structure in the generated ensemble exactly matches the KinCoRe labels of the ground-truth apo reference and 0 matches the KinCoRe labels of the ground-truth holo reference. This metric quantifies the tendency of the model to revert to the apo-like state despite the presence of the ligand.
- Both: At least one structure of the ensemble matches the KinCoRe labels of the ground-truth holo reference, and another matches the ground-truth apo reference. This metric quantifies the possibility that the holo-state could be recovered with more sampling or an effective ranking, which is considered a partial success.
- Neither: No structure in the generated ensemble exactly matches the KinCoRe labels of either the ground-truth holo reference or the ground-truth apo reference.

**Fig. 5:**
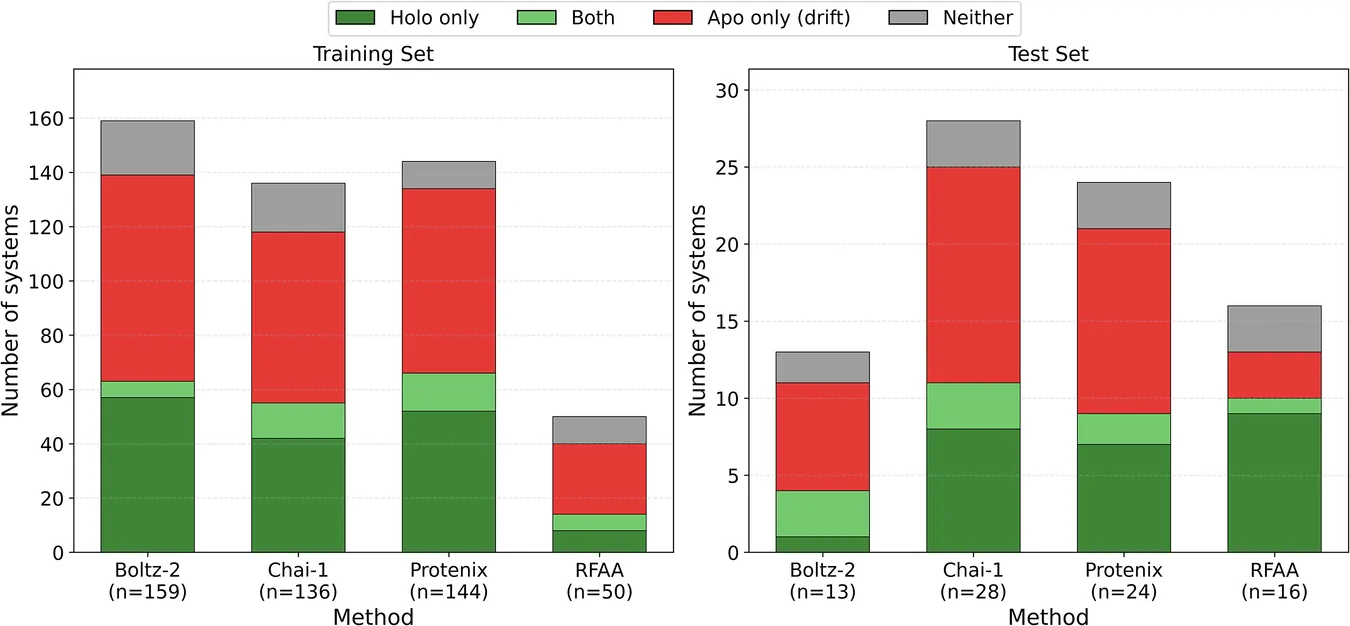
Generalization and apo-drift across co-folding models. Stacked fraction of systems in each holo/apo outcome category on holo inferences for Boltz-2, Chai-1, Protenix, and RFAA. Sample sizes per split (Train/Test systems): Boltz-2 159/13; Chai-1 136/28; Protenix 144/24; and RFAA 50/16.

A robustly generalizing cofolding model should keep a large holo fraction on both the training and testing data, whereas memorization shifts the predictions toward apo-like structures. As seen in Fig. 5, within the training set, the ‘holo-only’ and ‘both’ cofolding predictions represent roughly 40–45% of the kinase systems for Boltz-2, Chai-1, and Protenix, and ~30% for RFAA, indicating that the current cofolding models can sometimes rediscover the correct kinase conformational states. However, all four models display over 40% apo drift within the training set, suggesting that a large portion of the predictions indeed default back to apo structures even with the presence of a ligand. Therefore, although the specific ligand-kinase combinations are present in the models’ training data, the models remain prone to ignoring the protein conformational shift induced by the ligand, hinting that the apo-drift phenomenon could be driven by the reliance of cofolding models on evolutionary information or structural memorization^46^.

Moreover, when comparing the test set distribution in Fig. 5 against the training set, all cofolding predictions except those from RFAA further shift toward the assignment of ‘apo-only’ or ‘neither’ relative to the training systems. RFAA does not show the same train-to-test degradation as the diffusion models, but, given its consistently low holo-recovery fraction even on training systems, this statistic likely reflects uniformly weak performance rather than superior generalization. With only *N* = 16 RFAA test systems, we cannot determine whether the generalization gap is specific to diffusion-based models or reflects RFAA’s weak baseline.

## Discussion

Our benchmarking of four popular cofolding models highlights a tension between confident geometric poses and biophysically faithful kinase conformational states. Most models attain strong pocket-centric metrics on large portions of KinConfBench, yet functional KinCoRe agreement lags behind what raw lDDT or RMSD might suggest. Framing success purely through the pocket-based metrics therefore obscures a clinically relevant failure mode: the predicted kinase-ligand complex can ‘look’ bound while the kinase adopts the wrong conformational layout for the ligand at hand.

Ensemble behavior compounds the issue for all cofolding models. When additional samples are drawn, Top-20 accuracy gains indicate that correct kinase conformational states sometimes appear deeper in the ranking, but per-target histograms of the number of accurate predictions remain dominated by all-or-nothing outcomes rather than graded uncertainty. None of the four cofolding models eliminates the need for external checks when structure-activity-relationships involving induced fit or critical motions are required, as in MAP4K1/7M0M, where all models converge on the misfolded activation loop. This observation likely arises from the limited structural diversity we observe among correctly classified samples, as the ~0.1 Å or ~5° dispersion falls below even the fluctuation range of a single stable conformational state, far short of what distinct induced-fit basins would require.

The holo/apo paired analysis sharpens this diagnosis. The prediction of apo kinase states even with input of a holo kinase-ligand pair is shared across all models. After the training cutoff, we find that the holo state prediction rates fall and apo drift rises for each architecture except for the deterministic folding model RFAA, due to its poor baseline performance. Thus, the apo drift on seen complexes points to a plausible underlying cause: predictions are dominated by the canonical, high-prevalence prior of the training distribution rather than by ligand-specific induced fit. Closing this gap likely demands both better generative breadth for rare induced-fit basins and confidence objectives that reward state discrimination, rather than pocket-centric metrics alone.

At its core, drug discovery requires conformational state correctness rather than solely fold correctness or memorization of training instances. Predicting the general architecture of a kinase is biologically insufficient if the specific, ligand-induced functional conformation is wrong. KinConfBench is meant to keep that standard explicit as cofolding models continue to mature. Several directions would extend this benchmark toward biological realism, spanning from the evaluation protocol to the input to cofolding models.

To address the diversity collapse, a better sampling scheme that rescues the model from getting trapped in the local minima would likely be the immediate next step. Strategies like MSA sub-sampling, supplying different structural templates, or incorporation of dynamics data during training are reasonable steps forward to alleviate the sampling failure^16,36,47,48^.

Looking forward, the scope of kinase benchmarking could be expanded to encompass the intricate regulatory mechanisms that define this protein family in its real biological environment. Our current work focuses on the interaction between wild-type domains and non-covalent inhibitors, yet kinase signaling is tightly modulated by PTMs and somatic mutations. Future benchmarks should rigorously assess whether cofolding models can predict how phosphorylation at specific regulatory sites or drug-resistant mutations alter the conformational ensemble. Furthermore, the rise of covalent inhibitors in oncology necessitates a focused evaluation of how cofolding models handle the shift in protein context when a covalent bond is formed, as covalent ligands typically bring more unforeseen perturbations than simple non-covalent docking.

In addition to testing on known crystal structures, the ultimate test of a cofolding model’s biophysical reasoning will be its ability to simulate competitive dynamics and negative binders. In a physiological environment, kinases are rarely isolated with just a single ligand. For Type I kinase inhibitors, they constantly exist in a competitive environment where they must displace ATP or compete with other endogenous substrates. For confirmed non-binders, no genuine binding event exists to drive an induced-fit transition, so a physically faithful model should default to the apo conformation rather than fabricate a holo state. Future generations of benchmarks should evaluate the ability of models to handle multi-ligand scenarios, probing whether the presence of competing binders drives the protein into distinct conformational equilibria. In addition, the negative binders are abundant in high-throughput screening data, where binders typically constitute on the order of 1% of compounds tested^49^, yet remain almost entirely unexploited in structure-prediction benchmarks due to the lack of associated crystal structures. Moving beyond PDB-derived benchmarks to incorporate more realistic experimental data will be essential for evolving these tools from structural prediction engines into computational assays for drug discovery.

## Methods

### Cofolding model details and inferences

When performing inferences for Boltz-2^16^, Chai-1^14^, Protenix^17^, and RFAA^13^, we follow the official inference pipelines for each method to represent standard usage scenarios. Multiple sequence alignments (MSAs) are generated and cached with each tool’s default databases and pairing strategies. For Boltz-2, Chai-1, and Protenix, the ColabFold server^3^ is used for MSA generation, while sequence pairing follows the defaults of the respective model repositories. For RFAA, the default MSA pipeline supplied in their GitHub repository is used. Input sequences are restricted to the canonical kinase domain boundaries defined by UniProt, so the benchmark stresses domain-level cofolding behavior.

Inferences are performed on NVIDIA A100 GPUs across the 1420 unique configuration pairs (43 apo + 1377 holo) defined during curation. Post-inference, structures are passed through the Dunbrack KinCoRe annotation pipeline, and we exclude files that fail annotation because of malformed outputs. To ensure robust sampling of the conformational landscape, we generate *N* = 20 cofolding samples per target for each inference run. Output structures are saved in mmCIF format to ensure compatibility with the KinCoRe annotation pipeline. The final dataset produces 28,400 predictions for Boltz-2 and Protenix, 28,399 for Chai-1 (one predicted structure fails due to a parsing issue), and 28,300 for RFAA (five combinations fail because the ligand contains rare, unsupported elements).

### Kinase group classifications

To categorize the structural and functional diversity of the kinome, we utilize the standard Manning classification system^38^. The primary groups are defined as follows.

- AGC: Proteins including PKA, PKG, and PKC families.
- CAMK: Calcium/calmodulin-dependent protein kinases.
- CK1: Casein kinase 1 and closely related isoforms.
- CMGC: Proteins including CDK, MAPK, GSK3, and CLK families.
- STE: Homologs of yeast Sterile 7, Sterile 11, and Sterile 20 kinases.
- TYR: Tyrosine kinases.
- TKL: Tyrosine kinase-like proteins that are serine-threonine kinases.
- OTHER: Other kinases that don’t fall into the above classification.

### Details of KinCoRe annotations

Introduced by Modi and Dunbrack^37^, a kinase conformational label has the following eight annotations:

- Spatial Label: describes the position and orientation of the DFG motif as DFGin, DFGout, DFGinter, or None when key residues are not correctly mapped.
- Dihedral Label: captures the backbone and side chain dihedral angles of the X-DFG motif by using the Ramachandran region annotation (A, B, L, and E) for the X, D, and F residues and the DFG-Phe *χ*_1_ rotamer (minus= − 60°, plus= + 60°, and trans = 180°). These labels include BLAminus, BLBplus, ABAminus, BBAminus, BLBtrans, BLBminus, BLAplus, BABtrans, or None when key residues are not correctly mapped.
- *α*C-helix Label: indicates the placement of the *α*C-helix as either Chelix-in, Chelix-out, or None when key residues are not correctly mapped.
- Salt Bridge Label: denotes the formation of the conserved salt bridge as SaltBr-in, SaltBr-out, or None when key residues are not correctly mapped.
- N-term. Act. Loop Label: classifies the N-terminal region of the activation loop as ActLoopNT-in, ActLoopNT-out, or None when key residues are not correctly mapped.
- C-term. Act. Loop Label: classifies the C-terminal region of the activation loop as ActLoopCT-in, ActLoopCT-out, or None when key residues are not correctly mapped.
- Spine Label: indicates the assembly state of the regulatory spine as Spine-in or Spine-out, or None when key residues are not correctly mapped.
- Ligand Type: categorizes the bound ligand into Type 1, Type 1.5_Front, Type 1.5_Back, Type 2, Type 3, Allosteric, and No_ligand.

### Details of metrics for diversity analysis

- lys_glu_distance: Tracks the separation between the conserved *β*3-Lysine and *α*C-Glutamate, critical for evaluating *α*C-helix packing.
- saltbridge_distance: Monitors the formation and structural stability of the canonical salt bridge network.
- spine_distance: Measures the spatial arrangement of the hydrophobic residues comprising the regulatory spine, indicating its assembly state.
- dfg_hrd_distance: Evaluates N-terminal activation loop coupling by measuring the spatial separation between the DFG and catalytic HRD motifs.
- asp_phi: Captures the *ϕ* backbone dihedral angle of the DFG Aspartate residue.
- asp_psi: Captures the *ψ* backbone dihedral angle of the DFG Aspartate residue.
- asp_chi1: Describes the primary side-chain torsion (*χ*_1_) of the DFG Aspartate.
- asp_chi2: Describes the secondary side-chain torsion (*χ*_2_) of the DFG Aspartate. This is treated with twofold (“*π*”) symmetry so that flipped carboxylate placements are not penalized as opposite phases on the circle.
- phe_phi: Captures the *ϕ* backbone dihedral angle of the DFG Phenylalanine residue.
- phe_psi: Captures the *ψ* backbone dihedral angle of the DFG Phenylalanine residue.
- phe_chi1: Describes the primary side-chain torsion (*χ*_1_) of the DFG Phenylalanine.
- phe_chi2: Describes the secondary side-chain torsion (*χ*_2_) of the DFG Phenylalanine. Like the Aspartate, this is treated with twofold (“;*π*”) symmetry so that flipped aromatic substituent placements are not counted as opposite phases.

## Supplementary information

## Data Availability

All the inference results produced by four cofolding models and their associated analyses files for KinConfBench are available in Figshare: https://doi.org/10.6084/m9.figshare.31986663.

## Code availability

All codes used for KinConfBench curation, cofolding model inferences, and result analyses are available in GitHub: https://github.com/THGLab/KinConfBench.
