# Supplementary material for: A curated benchmark for cofolding models on kinase conformational states
Source: NPJ Drug Discov. 2026 Sep 2;3:33. doi: 10.1038/s44386-026-00068-z (PMC13538545; doi:10.1038/s44386-026-00068-z)
Supplement: Supplementary file 1 [file 44386_2026_68_MOESM1_ESM.pdf]

Supplementary Information
A Curated Benchmark for Cofolding Models on Kinase Conformational States

[1]Kenneth S. Pitzer Theory Center and Department of Chemistry, [2]Department of Bioengineering, [3]Department of Chemical and Biomolecular Engineering, University of California, Berkeley, CA, 94720 USA
† corresponding authors: kysun@berkeley.edu; thg@berkeley.edu

S1 Supplementary Tables **2**

S2 Supplementary Figures **6**

S1 Supplementary Tables

Table S1: **Percentage of KinCoRe spatial and dihedral label combinations for human kinases.** The definitions of Spatial and Dihedral Labels are defined in Methods. From the KinCoRe server PDB inventory (https://dunbrack.fccc.edu/kincore/home) and for KinConfBench (KCB) reference chains which focuses on a subset of high quality samples and filters out None labels during selection.

Spatial Label	Dihedral Label	All PDB	KinConfBench
DFGin	BLAminus	6806	1430
DFGin	BLAplus	316	27
DFGin	ABAminus	993	183
DFGin	BLBminus	433	96
DFGin	BLBplus	1008	238
DFGin	BLBtrans	255	102
DFGinter	BABtrans	26	5
DFGout	BBAminus	677	144
DFGin	None	836	0
DFGinter	None	224	0
DFGout	None	365	0
None	None	413	0

Table S2: **Distribution of KinCoRe spatial and dihedral labels for KinConfBench (KCB) and cofolding model predictions.** KCB percentages are derived from the 2,225-chain set described in the Results. Percentages for Boltz-2 ($n = 28,400$), Chai-1 ($n = 28,399$), Protenix ($n = 28,400$), and RFAA ($n = 28,300$) are calculated across 1,420 distinct kinase systems.

Spatial	Dihedral	KCB (%)	Boltz-2 (%)	Chai-1 (%)	Protenix (%)	RFAA (%)
DFGin	BLAminus	64.3	65.8	77.5	65.1	90.4
DFGin	BLAplus	1.2	1.1	1.2	1.5	0.1
DFGin	ABAminus	8.2	3.9	4.8	3.3	0.0
DFGin	BLBminus	4.3	3.0	2.0	3.4	5.3
DFGin	BLBplus	10.7	8.0	8.5	8.3	0.8
DFGin	BLBtrans	4.6	11.6	0.1	11.7	0.0
DFGin	None	0.0	0.4	0.4	0.2	1.3
DFGinter	BABtrans	0.2	0.0	0.0	0.0	0.0
DFGinter	None	0.0	0.8	0.6	0.1	0.4
DFGout	BBAminus	6.5	5.3	4.3	5.4	1.4
DFGout	None	0.0	0.1	0.5	0.1	0.2
None	None	0.0	0.0	0.1	1.0	0.1

Table S3: **Ground-truth KinCoRe spatial and dihedral label distribution over geometrically-correct cofolding predictions.** Each method column represents the label distribution over only the ligand-kinase combos where that method produced ≥ 1 geometrically-correct prediction. KCB represents the general distribution of labels for all chains from the KinConfBench data.

**Spatial**	**Dihedral**	**KCB (%)**	**Boltz-2 (%)**	**Chai-1 (%)**	**Protenix (%)**	**RFAA (%)**
DFGin	BLAminus	64.3	73.4	80.2	72.7	90.2
DFGin	BLAplus	1.2	0.9	1.0	0.9	0.3
DFGin	ABAminus	8.2	2.5	3.5	2.9	0.8
DFGin	BLBminus	4.3	2.3	2.0	2.2	1.8
DFGin	BLBplus	10.7	8.1	7.9	8.0	3.0
DFGin	BLBtrans	4.6	6.8	0.1	7.2	0.5
DFGin	None	0.0	0.0	0.0	0.0	0.0
DFGinter	BABtrans	0.2	0.0	0.0	0.0	0.0
DFGinter	None	0.0	0.0	0.0	0.0	0.0
DFGout	BBAminus	6.5	6.1	5.4	6.2	3.5
DFGout	None	0.0	0.0	0.0	0.0	0.0
None	None	0.0	0.0	0.0	0.0	0.0

Table S4: **152 systems where all four cofolding methods (Boltz-2, Chai-1, Protenix, RFAA) fail to produce a single prediction matching the ground-truth conformation.** Each entry is a KinConfBench system key (`GENE_Name_Ligands`).

System identifiers				
ABL1_HUMAN_66K	ACK1_HUMAN_1G0	ACK1_HUMAN_ACP	ACK1_HUMAN_DBQ	ACK1_HUMAN_LWX
ACK1_HUMAN_R7P	ACK1_HUMAN_T74	ACK1_HUMAN_T95	ACK1_HUMAN_WTP	ACK1_HUMAN_apo
ALK_HUMAN_25J	ALK_HUMAN_5P8	ALK_HUMAN_6YL	ALK_HUMAN_AWJ	ALK_HUMAN_HKJ
ALK_HUMAN_IV7	ALK_HUMAN_J3Y	AURKA_HUMAN_9YQ	AURKA_HUMAN_X6D	BRAF_HUMAN_54J
BRAF_HUMAN_FP3	BRAF_HUMAN_P06	BTK_HUMAN_YDA	CDK1_HUMAN_1QK	CDK1_HUMAN_F9Z
CDK2_HUMAN_09Z	CDK2_HUMAN_106	CDK2_HUMAN_2AN	CDK2_HUMAN_5JE	CDK2_HUMAN_89E
CDK2_HUMAN_A1D6S	CDK2_HUMAN_A27	CDK2_HUMAN_B49	CDK2_HUMAN_DTQ	CDK2_HUMAN_ES4
CDK2_HUMAN_HGH	CDK2_HUMAN_JWS	CDK2_HUMAN_LS1	CDK2_HUMAN_LS3	CDK2_HUMAN_LS4
CDK2_HUMAN_N5R	CDK2_HUMAN_0W6	CDK2_HUMAN_R0N	CDK2_HUMAN_TIY	CDK2_HUMAN_X06
CDK2_HUMAN_X35	CDK2_HUMAN_X44	CDK2_HUMAN_Y8L	CDK2_HUMAN_Z19	CDK2_HUMAN_Z63
CDK5_HUMAN_65L	CDK7_HUMAN_WZ8	CDK8_HUMAN_6A7	CDK8_HUMAN_apo	CHK1_HUMAN_306
CHK1_HUMAN_373	CHK1_HUMAN_76A	CHK1_HUMAN_AGY	CHK1_HUMAN_H0K	CHK1_HUMAN_YM5
CHK1_HUMAN_YM6	CLK1_HUMAN_WAZ	CSK21_HUMAN_4B0	CSK21_HUMAN_A1H6F	CSK21_HUMAN_EXX
DAPK1_HUMAN_A1L2V	DAPK1_HUMAN_BD4	DAPK1_HUMAN_HCC	DAPK1_HUMAN_LU2	DAPK1_HUMAN_PIT
DAPK1_HUMAN_STU	DAPK3_HUMAN_4RB	DAPK3_HUMAN_U7E	DAPK3_HUMAN_apo	DCLK1_HUMAN_XBD
DCLK1_HUMAN_apo	EGFR_HUMAN_26X_ATP	EGFR_HUMAN_ANP_IXR	EGFR_HUMAN_KY9	EGFR_HUMAN_W2R
EPHA2_HUMAN_92Q	EPHA2_HUMAN_DXK	EPHA2_HUMAN_L66	EPHA2_HUMAN_WT3	ERN1_HUMAN_4L5
ERN1_HUMAN_ADP	IGF1R_HUMAN_PDR	KCC2D_HUMAN_K88	KIT_HUMAN_K3R	KSYK_HUMAN_1B6
KSYK_HUMAN_X7G	LCK_HUMAN_9NH	LIMK1_HUMAN_RXN	M3K5_HUMAN_NJV	M3K5_HUMAN_STU
M4K1_HUMAN_2WI	M4K1_HUMAN_A1AP0	M4K1_HUMAN_A1BWM	M4K1_HUMAN_ANP	M4K1_HUMAN_YK1
MET_HUMAN_MDI	MK01_HUMAN_1FM	MK01_HUMAN_2SH	MK01_HUMAN_33A	MK01_HUMAN_35X
MK01_HUMAN_362	MK01_HUMAN_360	MK01_HUMAN_ANP	MK01_HUMAN_F29	MK01_HUMAN_FRZ
MK07_HUMAN_4QX	MK07_HUMAN_R4L	MK14_HUMAN_GK1	MK14_HUMAN_LI4	MK14_HUMAN_P0F
PAK1_HUMAN_40Q	PAK1_HUMAN_40R	RIPK1_HUMAN_Q1A	ROCK1_HUMAN_07Q	ROCK1_HUMAN_07R
ROCK1_HUMAN_0HD	ROCK1_HUMAN_3J7	ROCK1_HUMAN_3NC	ROCK1_HUMAN_3ND	ROCK1_HUMAN_4KH
ROCK1_HUMAN_4KK	ROCK1_HUMAN_4TW	ROCK1_HUMAN_65R	ROCK1_HUMAN_6U1	ROCK1_HUMAN_6U2
ROCK1_HUMAN_8UV	ROCK1_HUMAN_ANP_GTP	ROCK1_HUMAN_B4J	ROCK1_HUMAN_B4V	ROCK1_HUMAN_B4Y
ROCK1_HUMAN_B5G	ROCK1_HUMAN_H52	ROCK1_HUMAN_HFS	ROCK1_HUMAN_J0P	ROCK1_HUMAN_M77
ROCK1_HUMAN_VFS	ROCK1_HUMAN_Y27	ROCK2_HUMAN_5YS	ROCK2_HUMAN_YEW	ROCK2_HUMAN_Z80
SRPK1_HUMAN_RXZ	TTBK1_HUMAN_apo	VRK2_HUMAN_7DZ	VRK2_HUMAN_KJD	WEE1_HUMAN_99J
WEE1_HUMAN_99M	WEE1_HUMAN_99V			

S2 Supplementary Figures

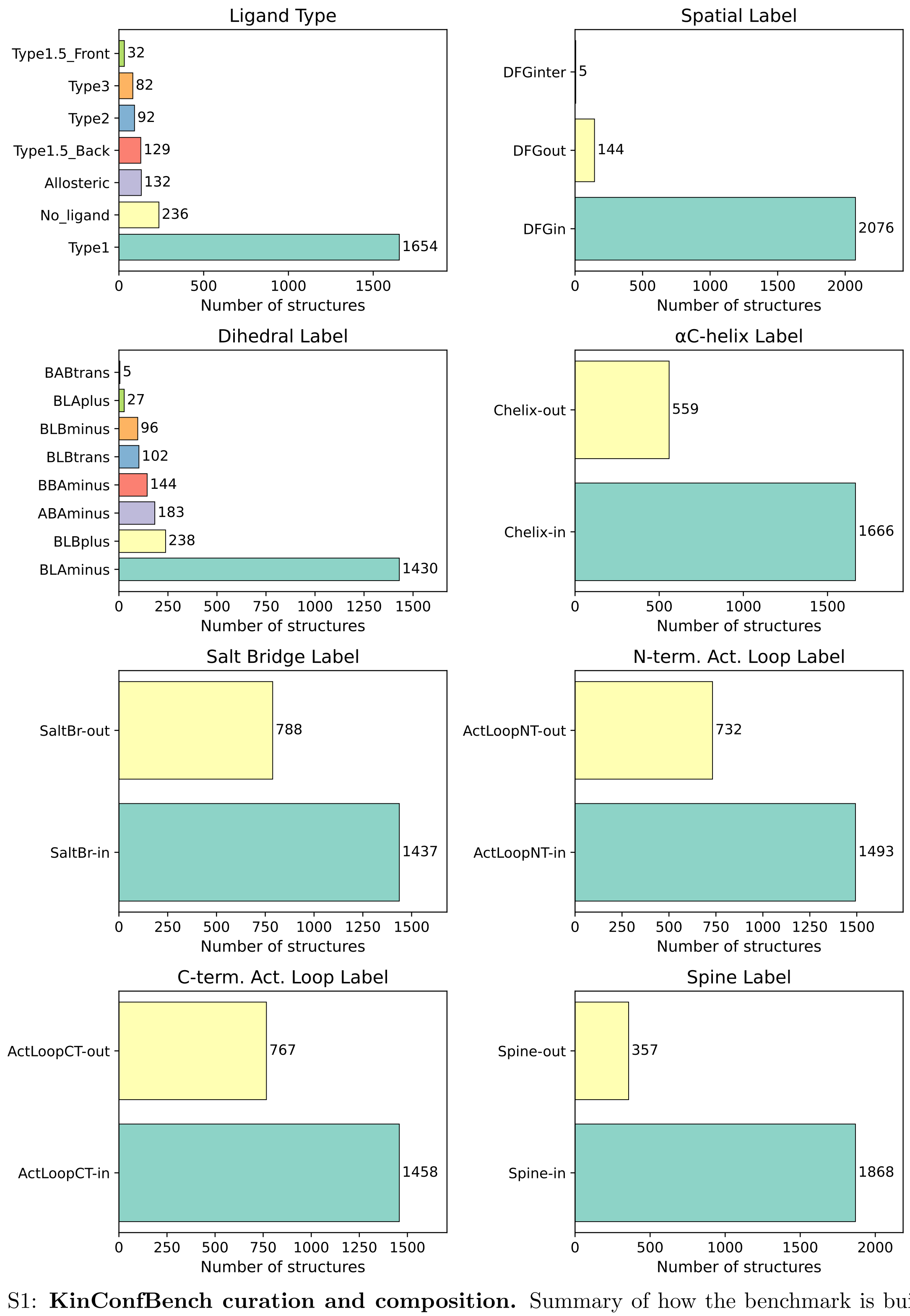

Figure S1: **KinConfBench curation and composition.** Summary of how the benchmark is built from PDB-derived kinase chains: counts by holo versus apo, single- versus multi-ligand complexes, redundancy filtering, and the distribution of entries across Manning kinase groups and ligand/chemotype categories. Together these panels document that the final benchmark is holo-heavy (reflecting the PDB) yet retains diverse genes and chemotypes, and that reported model statistics are computed on the curated non-redundant set rather than the raw PDB pull.

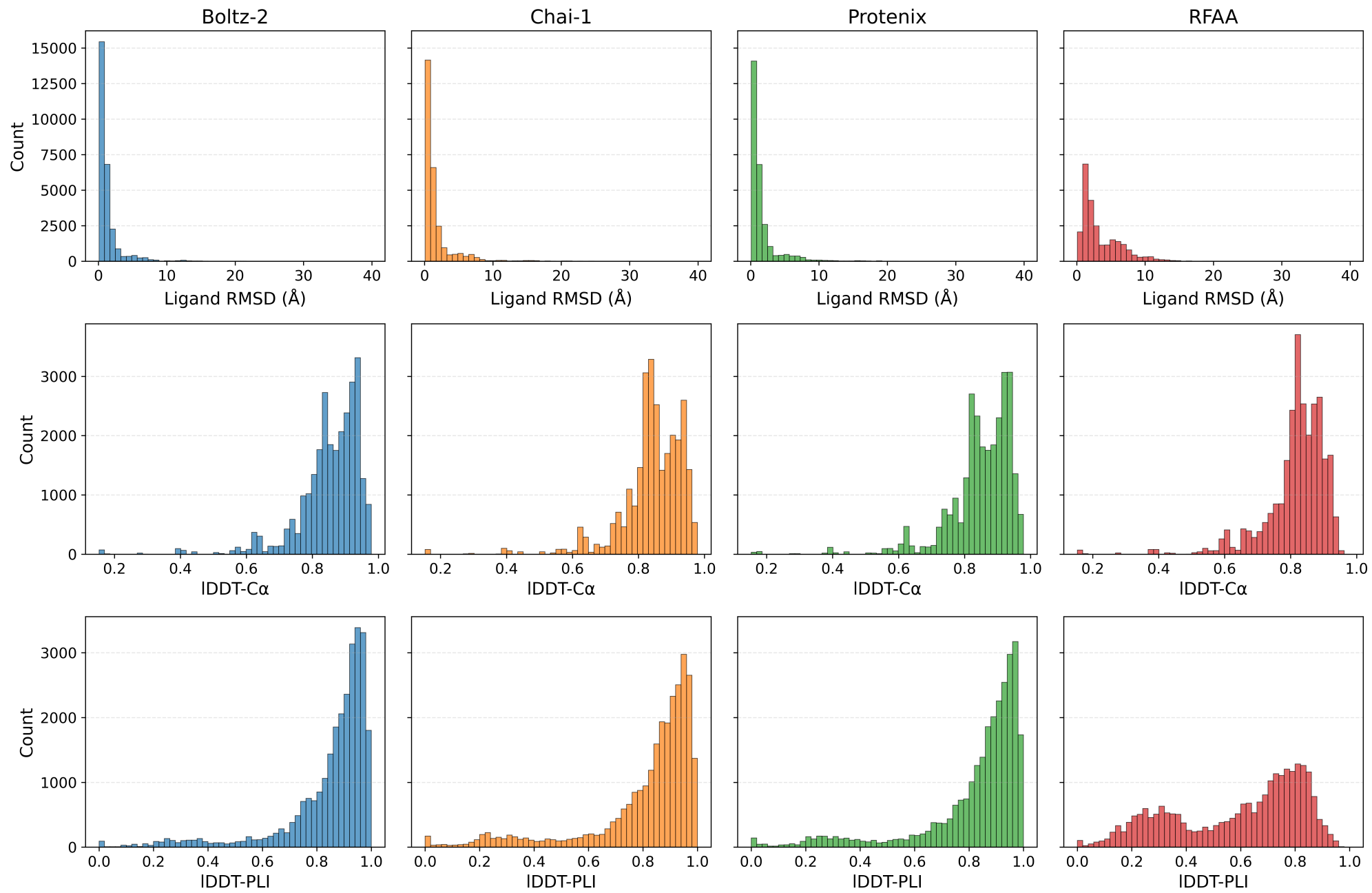

Figure S2: **Distribution of geometric metrics for cofolding predictions.** Distributions of ligand heavy-atom RMSD, lDDT-Cα, and lDDT-PLI for all ensemble predictions.

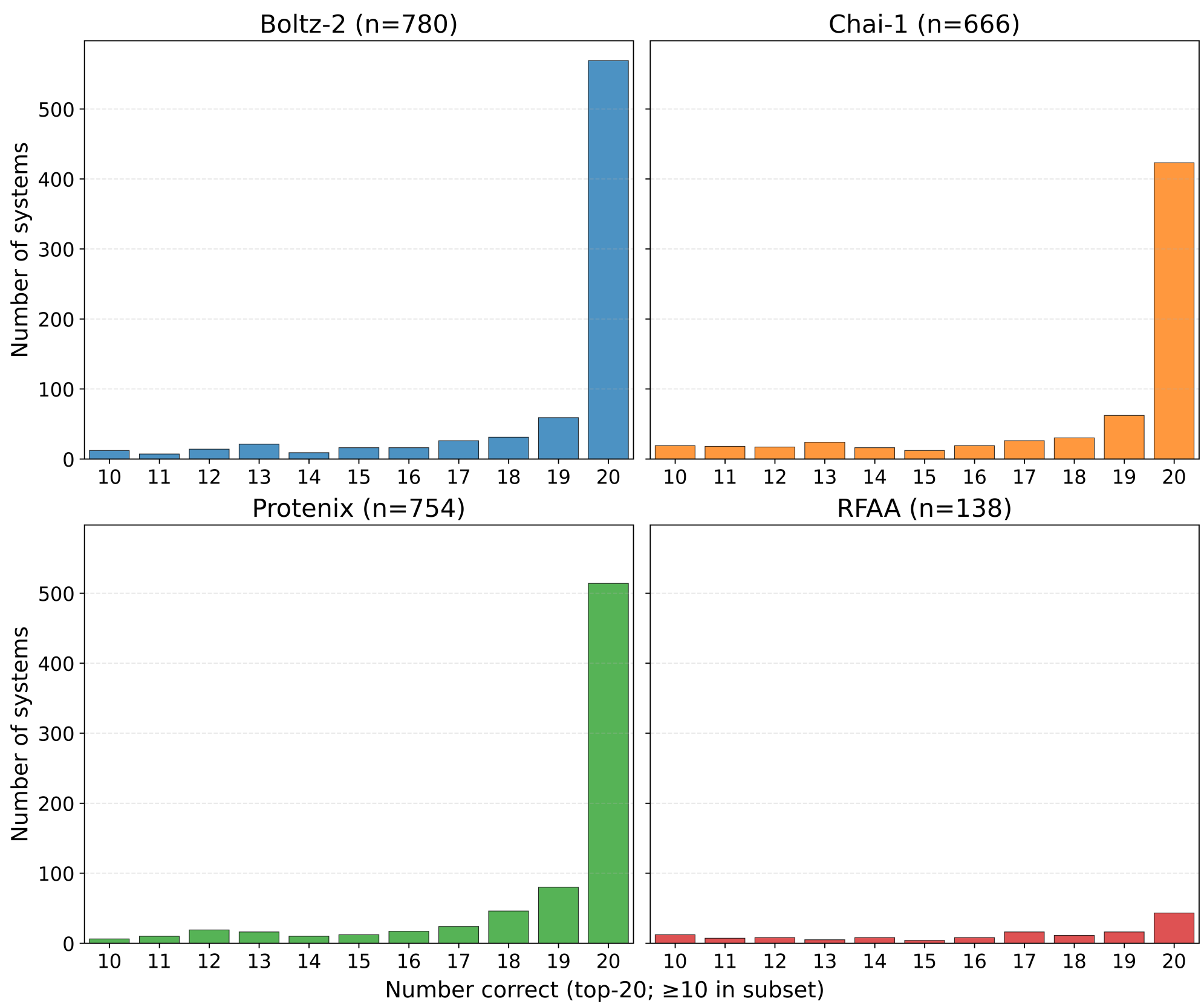

Figure S3: **Correct-prediction counts on the diversity subset.** Same quantity as in Figure 2(c), but restricted to the subset used for ensemble diversity analysis, i.e. systems where each cofolding method has at least ten all-labels-correct predictions in the top-20.

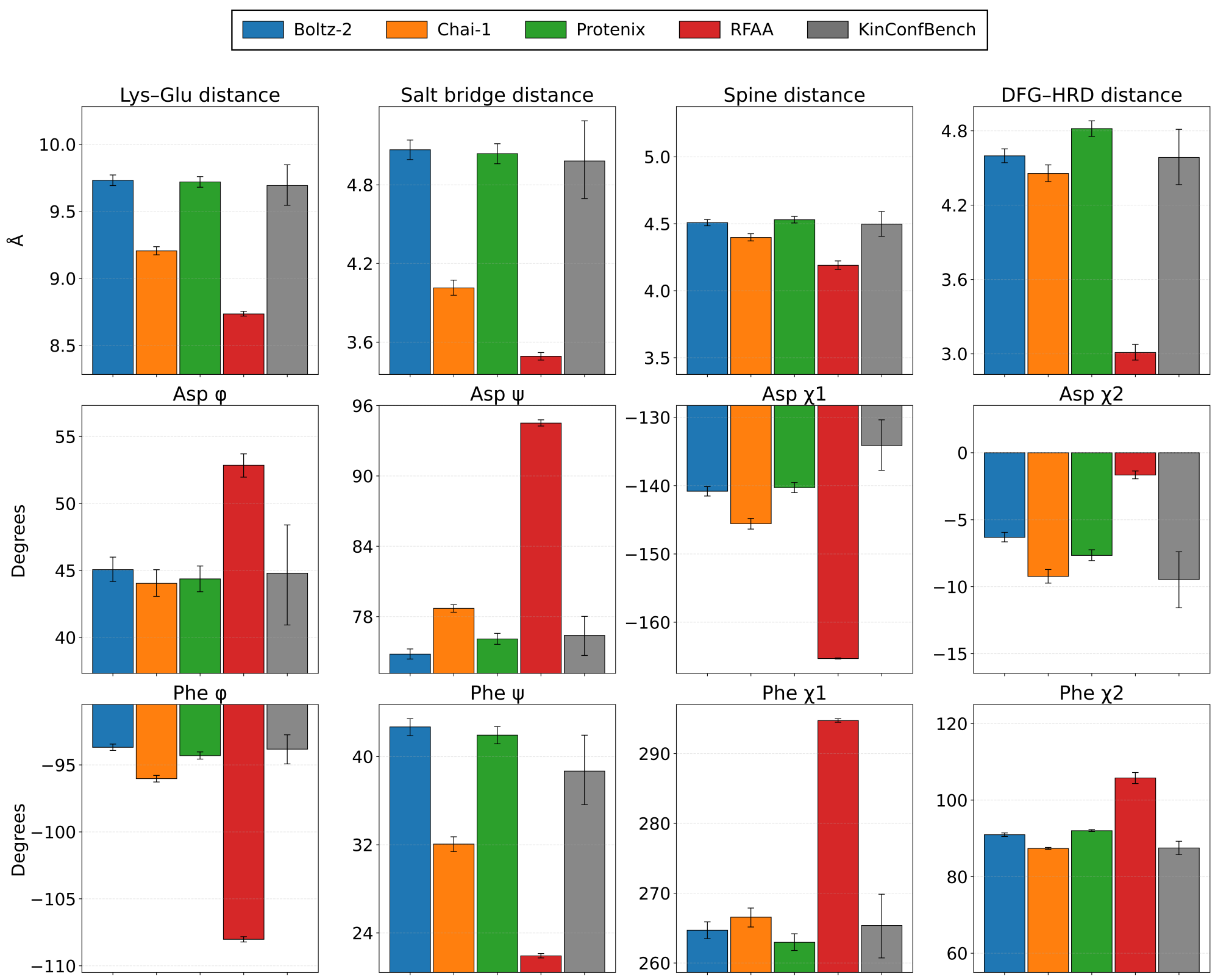

Figure S4: **Mean of key distance and angle metrics for cofolding predictions and KinConfBench.** The plot displays mean values for Boltz-2 (blue), Chai-1 (orange), Protenix (green), and RFAA (red) predictions, with KinConfBench PDB reference values provided in gray for comparison. Error bars are bootstrap 95% confidence intervals for each pooled mean.

References
